# Supplementary material for: The effect of tranexamic acid on intraoperative blood loss in patients undergoing brain meningioma resections: Study protocol for a randomized controlled trial
Source: PLoS One. 2023 Aug 31;18(8):e0290725. doi: 10.1371/journal.pone.0290725 (PMC10470952; doi:10.1371/journal.pone.0290725)
Supplement: S2 File — (DOC) [file pone.0290725.s002.doc]

Project number: PX-2022018

**Efficacy of tranexamic acid in patients undergoing huge meningioma resection**

**Project name**: Efficacy of tranexamic acid in patients undergoing huge meningioma resection

**Principal Investigator & Department**: Shu Li/Department of Anesthesiology, Beijing Tiantan Hospital, Capital Medical University

**Sponsor**: Beijing Municipal Hospital Administration Center

**Institution of principal investigator:** Beijing Tiantan Hospital, Capital Medical University

**Study period**: January 2022－December 2024

**Version**: V1.1.1

**Version date**: 2022.05.09

**Protocol Abstract**

| **Project name** | Efficacy of tranexamic acid in patients undergoing huge meningioma resection |
| --- | --- |
| **Objectives of research** | 1. To investigate the effect of tranexamic acid (TXA) on blood loss during the resection of huge meningioma. 2. To investigate the effects of TXA on coagulation function, blood salvage, transfusion of allogeneic blood products in patients undergoing huge meningioma resection; to investigate the effect of TXA on the resection of huge meningioma and the hemostasis of surgical field after resection.   3、To investigate the effect of TXA on epilepsy and other adverse events after resection of huge meningioma.  4、To investigate the effects of TXA on length of stay, ICU stay and hospital charge of patients after resection of huge meningioma. |
| **Study design** | Prospective, single-center, randomized, controlled, double-blinded trial |
| **Sample size** | 228 cases |
| **Study population** | Inclusion criteria ***Preoperative computed tomography (CT), or magnetic resonance imaging (MRI) suggested supratentorial meningioma with diameter no less than 5cm in any of three axes.*** 2）Patients aged between 18 and 65 years.  3）American Society of Anesthesiologist (ASA) physical status Ⅰ to Ⅲ.  4）Written informed consent obtained. |
|  | Exclusion criteria   1. Allergic to TXA. 2. With a history of thrombotic disease. 3. Under anticoagulant or antiplatelet therapy. |
| **Treatment protocol** | After confirming that the patients meeting the inclusion criteria, patients were randomly assigned to one of 3 groups: high-dose group, low-dose group and control group. The high-dose group was given a loading dose of 20mg/kg TXA within 10 minutes after induction, followed by a continuous infusion of TXA at a rate of 5mg/kg/hour. Low-dose group received a loading dose of 20mg/kg TXA within 10 minutes, and then continued infusion of identical volume of normal saline. Control group was given identical volume of normal saline. Follow-up will be conducted during the operation, 1-5 days after operation, and 6 months after operation to collect data. |
| **Outcome and safety measures** | Primary outcome: the estimated cumulative intraoperative blood loss  Secondary outcome:   1. Intraoperative coagulation function. 2. Calculated blood loss. 3. Intraoperative cell salvage use and allogeneic blood products use. 4. Simpson grading scale for the extent of meningioma resection. |
|  | Safety measures:   1. Frequency and number of postoperative seizures. 2. Incidence of other intraoperative complications, including drug allergy, intraoperative hypoxemia, refractory hypotension, and pulmonary embolism and so on. 3. Incidence of other postoperative complications, including deep vein thrombosis, perioperative cerebral ischemia, intracranial hematoma, hydrocephalus, infection, acute renal injury, and acute myocardial injury. |
|  | Economic measures:   1. ICU occupancy rate, and the length of ICU stays. 2. The length of hospital stays, and total hospitalization cost. |
| **Statistical analysis plan** | Primary outcome: The Student’s t-test. Secondary outcome: Descriptive statistics will be reported as means with standard deviation and medians with interquartile range for normally distributed data and skewed continuous data, respectively, and counts (percentage) for categorical data. The Student’s t-test will be used to compare normal distribution data and the Mann-Whitney U test for skewed data. The Chi-square test will be used for categorical data. Event time data will be analyzed using Kaplan-Meier survival analysis, and differences among groups were assessed using log-rank test and proportional risk regression models. |
| **Study period** | January 2022－December 2024 |

1. **Background**

Meningiomas are the most common intracranial tumors, accounting for more than 30%[1]. Although the nature of meningioma is mostly benign, due to its occupying effect and invasion of blood vessels, meningioma patients are often accompanied by severe intracranial pressure elevation, encephaledema, and cranial nerve injury, and thus lead to serious neurological dysfunction. Huge meningiomas, defined as diameter ≥5cm, account for about 11% of primary intracranial neoplasm[2]. surgical resection of huge meningiomas is the preferred treatment to improve neurological outcomes. However, most of the huge meningiomas are accompanied by a rich blood supply, compressing and/or distorting blood vessels in their growth process. Some of the meningiomas also surround peripheral blood vessels and involve the venous sinuses, invading the scalp and skull, and the blood loss during resection could be as high as 4000mL[2]. Besides that, the tissue plasminogen activator in tumor and peritumoral brain tissues can induce fibrinolysis and contribute to intraoperative hemorrhage[3]. Massive intraoperative bleeding and severe hemodynamic fluctuations during the resection of huge meningioma may lead to organ injury, coagulopathy and even be life-threatening[4, 5]. Therefore, perioperative blood management of huge meningioma resection is challenging.

There are many techniques for perioperative blood management in neurosurgery, but each blood management technique is accompanied by certain deficiencies and a heavy economic burden. Even with all of the above blood management methods, allogenic transfusion is inevitable in some cases due to excessive blood loss, while allogenic transfusion presents a high risk of immunosuppression, hemolysis, postoperative infection, and transfusion-related lung injury, resulting in prolonged hospital stay and even death[6-10].

Tranexamic Acid (TXA) is a synthetic antifibrinolytic drug with a lysine-like structure. TXA can inhibit plasminogen activation and prevent plasminogen from binding to fibrin, thus blocking the fibrinolytic process, preventing thrombolysis, reducing bleeding, and reducing the blood transfusion rate[11, 12].

TXA has been widely used in trauma, otolaryngology, obstetrics, cardiac surgery, and orthopedics,[13-20] whether TXA can effectively reduce intraoperative bleeding and blood product infusion in patients undergoing neurosurgical meningioma surgery without increasing perioperative adverse events remains to be elusive. Previous studies have shown that TXA can significantly reduce blood loss in neurosurgeries (830ml vs 1124ml, p=0.03). However, patients in this study had a variety of tumor locations, including the skull base, parasella, subtentorial, parasagittal sinus, etc., and tumor sizes also varied. It is unclear whether TXA can reduce blood loss in patients undergoing huge meningioma resection, who are with a high risk of massive bleeding.

Therefore, we hypothesize that TXA with different doses can effectively reduce intraoperative blood loss in patients undergoing huge meningioma resection.

1. **Objectives**

**1. Primary objective:** To investigate the effect of TXA on blood loss during the resection of huge meningioma.

**2.** **Secondary objective:**

1) To investigate the effects of TXA on coagulation function, surgical field blood recovery, and use of allogeneic blood products in patients undergoing huge meningioma resection.

2) To investigate the effect of TXA on the resection of huge meningioma and the bleeding in the operative field after resection.

3) To investigate the effect of TXA on epilepsy and other adverse events after resection of huge meningioma.

4) To investigate the effects of TXA on length of stay, ICU stay and hospitalization cost of patients after resection of huge meningioma.

1. **Study design and trial procedures**

**1. Study design**

1）Study design: The study is a prospective, randomized, controlled, double-blinded trial.

2）Randomization: Using computer software to generate random sequences, patients were randomly assigned to one of three groups (high-dose, low-dose or control group）based on the grouping of random numbers with a 1:1:1 ratio.

3）Blinding: The participants, responsible anesthesiologists, neurosurgeons, and outcome assessors will all be blinded to the allocation until the completion of the study analysis.

4）Research center: Department of Anesthesiology, Beijing Tiantan Hospital, Capital Medical University.

5）Sample size: 228 cases.

6）Rationality and basis of indications: Currently, clinical studies on TXA are mainly limited to orthopedics, obstetrics, cardiac surgery, trauma, etc., and there are few studies in neurosurgery. However, huge meningioma is prone to massive intraoperative bleeding due to its huge tumor volume, rich blood supply, vascular invasion, and cranial nerve damage caused by the tumor. Previous randomized controlled trials of the effect of TXA on intraoperative blood loss in meningioma found that TXA significantly reduced intraoperative blood loss. However, the study sample size was small, and the tumor locations were highly heterogeneous, so the results of these studies cannot be applied to the population of huge meningioma. The purpose of this study is to investigate the effect of TXA on intraoperative blood loss in meningiomas with a diameter over 5cm. At present, our institution has confirmed the safety of TXA in regard to postoperative seizure in patients undergoing meningioma resection, and the efficacy of TXA needs to be further investigated.

**2. Sample size and study project**

According to previous studies, the intraoperative blood loss for huge meningioma was about 1000ml, and the overall standard deviation of the population was about 400ml. A single TXA infusion was assumed to be effective in reducing blood loss by about 25%, and continuous TXA infusion could further reduce blood loss by 25%, so the minimum effect size was 250ml. A sample size of 228 patients (76 per group) is calculated to provide 90% power to detect the between-group difference at a significance level 𝛼 of 0.017 and the overall type I error of 0.05, with a dropout rate of 2.5% with Bonferroni adjustment.

**3. Study period**

January 2022－December 2024.

**4. Study population**

The plan is to consecutively recruit patients undergoing elective huge meningioma resection at Beijing Tiantan Hospital, Capital Medical University from January 2022 to December 2024.

**Inclusion criteria:** Patients undergoing elective resection of huge meningioma, and preoperative imaging computed tomography (CT), or magnetic resonance imaging (MRI) suggested meningioma no less than 5cm in diameter in any of three axes; patients aged between 18 and 65 years; ASA physical status Ⅰ to Ⅲ; get written informed consent.

**Exclusion criteria:** Allergic to TXA; with a history of thrombotic disease; under anticoagulant or antiplatelet therapy; informed consent cannot be obtained.

When a serious adverse event (SAE) occurs during the trial, we will closely monitor the event until it is resolved and stabilized. The SAE would be immediately reported to the relevant authorities, and the principal investigator would be notified to determine the severity and consequence. In case of intraoperative adverse events such as severe allergy to the research drug, the infusion of the research drug would be stopped according to the judgment of the anesthesiologist.

1. **Study method**
2. **Blinding:** Study participants, anesthesiologists, and post-operative outcome assessors will be blinded to randomization. Randomization code will be prepared in opaque envelopes, and randomization will be concealed until the primary outcome assessment of the last patients is accomplished. If there was a life-threatening SAE during the intervention, the principal investigator would decide whether to unblind the case and this participant would be dropped out. TXA and placebo will be prepared by an individual investigator who is blinded to allocation, this investigator will also label the container "research drug". This investigator will not participate in the intervention or any assessment and follow-up.
3. **Grouping and intervention:** Participants were randomly divided into three groups: low-dose group, high-dose group, and placebo (control group). Intravenous infusion of TXA or placebo (saline) will be initiated after anesthesia induction. The research drug is TXA manufacturer in 2mL with 200mg. Patients in the high-dose group will receive an intravenous infusion of 20mg/kg TXA followed by a continuous infusion of 5mg/kg/h until the end of the operation. Patients in the low-dose group will receive 20mg/kg intravenously TXA only. Patients in the control group will receive an identical volume of 0.9% normal saline. Both TXA and saline for a single infusion were stored in a 100ml container sheltered from light, and the infusion rate was 250ml/h.
4. Standard anesthesia management: After establishing reliable venous access, standard monitoring, including routine electrocardiograph (ECG), non-invasive blood pressure, pulse oxygen saturation, end-tidal carbon dioxide, and bispectral index (BIS) monitoring will be initiated. After pre-oxygenation for 6min, standardized anesthesia induction will be initiated with propofol 1-2mg/kg or etomidate 0.2mg/kg, sufentanil 0.2-0.4ug/kg, rocuronium 0.9mg/kg or cisatracurium 0.2mg/kg. The actual dose can be adjusted according to the patient's physiological condition. Intraoperative anesthesia can be maintained by intravenous anesthesia or combined with intravenous anesthesia, and the intraoperative BIS will be maintained between 40 and 60. H3 receptor blocker (Tropisetron 5mg) will be administered before the end of surgery to prevent nausea and vomiting. All medications will be recorded in detail, and patients will be transferred to the ward or post-anesthesia care unit (PACU) or ICU. Patient-controlled analgesia devices were routinely used after surgery, and sufentanil was selected as the analgesic formulation. The dosage was determined by the anesthesiologist in charge. If the subject still presented severe postoperative pain (visual analogue scale (VAS) ≥5, with agitation), other rescue analgesic drugs will be administered by the physician in charge and be recorded in detail.
5. **Concomitant medication**

This study focuses on clinical anesthesia, anesthesia drugs can be concomitant drugs.

1. **Trial procedures**

**Enrollment:** 1）Whether it meets the inclusion criteria; 2) Whether it meets the exclusion criterion; 3) Informed consent; 4) Randomization.

**Peri-operation:** Baseline characteristics, family history, medical history, physical examination, preoperative lab test (blood routine, coagulation function and chemistry panel), preliminary radiology diagnosis, pre-operative evaluation (GCS score, Charlson complications score, ASA classificaiton, Karnofsky score, Caprini thrombosis risk score).

**Intraoperative:** 1) target mean arterial pressure; 2) cranial nerve block; 3) use of positive inotropic drugs or vasopressor; 4) average inhaled oxygen concentration; 5) coagulation; 6) arterial blood gas (ABG) test; 7) estimated blood loss; 8) type and volume of allogenic transfusion; 9) intraoperative adverse events; 10) tumor resection extension; 11) surgical hemostasis score and hemostasis difficulty; 12) use of topical hemostatic agents; 13) anesthetic drugs; 14) anesthetic parameters; 15) physiological parameters.

**PACU：**1) time of PACU admission; 2) PACU events; 3) drug use; 4) time of PACU discharge.

**Day 1**: 1) outcomes: assessment time, KPS score, GCS score, postoperative epilepsy, embolism, infection, postoperative hematoma, re-operation, ICU admission, death, other adverse events, and assessment of neurological dysfunction; 2) laboratory test; 3) assessment of thrombosis risk by Caprini score.

**Day 3**: outcomes: assessment time, postoperative epilepsy, embolism, infection, postoperative hematoma, re-operation, ICU admission, death, other adverse events, and assessment of neurological dysfunction.

**Day 5±2**: 1) outcomes: assessment time, death, postoperative epilepsy, embolism, infection, postoperative hematoma, re-operation, ICU admission, other adverse events, and assessment of neurological dysfunction; 2) Brice Awareness Questionnaire 5±2 days after surgery; 3) postoperative brain image and EEG.

**Discharge:** 1) pathology diagnosis; 2) discharge summary (date of discharge, recovery, length of stay, referral, length of stay in ICU, hospital charges, epilepsy medication); 3) postoperative treatment plan; 4) clinical assessment (anemia, epilepsy, embolism, infection, hematoma, re-operation, blood transfusion, etc.).

**180 days**: 1) outcomes: assessment time, death, KPS score, GCS score, postoperative epilepsy, embolism, infection, postoperative hematoma, re-operation, ICU admission, other adverse events, and assessment of neurological dysfunction.

1. **Follow-up period**

**Follow-up period:** the day before surgery, 1, 3, 5±2 and 180 days after surgery.

**Follow-up items:** examination (blood routine, coagulation function and chemistry panel) on the first day after surgery, Caprini risk score on the first day after surgery, KPS score on postoperative day 1, day 3, day 5 ±2 and at discharge and on 180 days. Others include GCS score and complication evaluation.

1. **Outcome assessments**

The primary outcome is the estimated cumulative intraoperative blood loss, which is calculated using the formula: collected blood volume in the suction canister (mL) – the volume of flushing (mL) + the volume from the gauze tampon (mL).

The secondary outcomes include:

1. Intraoperative coagulation function monitoring assessed by rapid TEG at the beginning and the end of the operation.
2. Calculated blood loss: Calculated blood loss = estimated blood volume×(Hct_i_−Hct_f_)+transfused RBC volume]/Hct_mean_.
3. Intraoperative cell salvage use and allogeneic blood products use. The indications for routine blood transfusion in our institution are as follow:

**a.** Hb < 9g/dl or Hct < 30%. If the intraoperative pathology consultation indicated a WHO classification of II or I, we will first consider cell salvage. If intraoperative pathology was unavailable or WHO classification > II, allogeneic blood transfusion will be preferred.

**b.** Use of frozen plasma, cryoprecipitate, and platelet products, will be initiated based on the American Society of Anesthesiologists Task Force on Blood Component Therapy: PT/APTT is more than 1.5 times prolonged, fibrinogen <1g/L, or diffuse microvascular bleeding at the surgical field (although there is no surgical hemostasis and/or no visible clots in the surgical field).

1. Simpson grading system for the extent of meningioma resection.
2. Hemostasis: Hooda score.
3. Operative field surgical hemostasis score using VAS score.

Safety outcome includes 1) frequency and episodes of postoperative seizure; 2) other intraoperative complications (allergy, hypoxemia, refractory hypotension, pulmonary embolism, etc.) other postoperative complications (venous thrombosis, new cerebral ischemia, intracranial hematoma, hydrocephalus, infection, abnormal renal function, myocardial infarction).

Economic outcome: 1) ICU admission, length of ICU stays; 2) length of hospital stay and hospital charge.

1. **Potential risks, benefits of the project & risk management**

Adverse effects of TXA will be closely monitored from the beginning of the research drug infusion through the fifth postoperative day. Investigators will record all adverse events, including type, time of occurrence, duration, and prognosis. The intraoperative anesthesiologist has an obligation to stop the infusion and record the cause. All adverse events will be closely monitored until a stable state is reached. The principal investigator will be informed of any SAEs and determine the severity and causation of these events. All adverse events associated with the study will be recorded and submitted to the Ethics Committee in the annual report. The principal investigator will be responsible for adverse events.

According to the literature review, the main adverse event of TXA is epilepsy, followed by embolic events. When adverse events occurred, emergency measures will be initiated immediately, including sedation to control epilepsy, vasodilator, and fluid therapy to reduce the occurrence of ischemic events.

1. **Quality control**
2. Responsibility of investigator

The investigator should follow the research protocol, the Good Clinical Practice guideline and relevant laws and regulations.

The investigator should follow the study procedure (including safety measures) in the investigation procedure. The investigators should accurately and clearly provide standardized and reliable data and information required in the clinical trial protocol and ensure that the follow-up team has direct access to the original data.

The principal investigators may appoint the appropriate persons to be assistants. The assistants will help in the clinical trial management according to the protocol. All assistants will be supervised by the principal investigator. They will be provided with trial protocol and all necessary information. The sponsor of the trial is Beijing Municipal Administration of Hospitals Incubating Program. The program will monitor and manage the trial in appropriate means, including ethic committee review, audit protocol compliance, and data quality control.

1. Audit

The primary concern of the audit team is to ensure that the trial is ethical, scientific, professional, and standardized. In accordance with the GCP principle, the audit team shall check the paper case report form (CRF) against the original data.

The audit team will assess the research progress, ensure the investigator and patient follow the protocol, and resolve urgent issues through regular site visits, letters, or phone calls. During these visits, the audit team and the principal investigator will jointly supervise the following key points: informed consent, recruitment and follow-up, and documentation and reporting of SAEs.

1. **Data preservation**

All data will be recorded in the paper CRF, which will be kept in the department's locked filing cabinet for at least 5 years. Both original and nature language data are encoded for data storage, review, tabulation, and analysis. The data will be securely entered and stored in an electronic database. Double data entry will be used.

1. **Data monitoring committee**

The Data Monitoring Committee (DMC) will be composed of experts in anesthesiology, ethics, statistics and methodology, as an independent body to monitor the safety, efficacy, ethical issues, and progress of the trial. The DMC will conduct audits through regular interviews or telephone calls. DMC reserves the right to review recruitment process at any time. The audit process will be independent.

1. **Statistics**

Sample size and statistical power: According to previous research reports, the intraoperative blood loss for huge meningioma was about 1000ml, and the standard deviation of the population was about 400ml. A single TXA infusion effectively reduced the amount of blood loss by about 25%, and continuous TXA infusion further reduced the amount of blood loss by 25%. Therefore, the estimated effect size is 250ml. A sample size of 228 patients (76 per group) is calculated to provide 90% power to detect the between-group difference at a significance level 𝛼 of 0.017 and the overall type I error of 0.05, with a dropout rate of 2.5% with Bonferroni adjustment.

1. Statistical analysis principle: Intention-to-treat analysis.
2. The Student’s t-test will be used to compare the primary outcome between groups, and a p-value <0.017 will be considered as a significant difference between the two groups. The overall p-value<0.05 was considered as a significant difference in blood loss among the three groups.
3. The Student’s t-test will be used for comparison between groups of normal distribution data of secondary outcome, and the Mann-Whitney U test will be used for skewed distribution data. The chi-square test was used to compare the categorical data. The primary outcome of the study is intraoperative blood loss, which is a continuous variable and it will be analyzed using the Student’s t-test or Mann-Whitney U test. For comparison between groups, Bonferroni correction is used to adjust P value.
4. **Ethical consideration**

This research will follow the relevant regulations, including the Declaration of Helsinki. Participant recruitment will be initiated only after the protocol is approved by the Ethics Committee. Before each subject is enrolled in the study, the investigator will introduce participant or his/her surrogate the purpose, procedure and possible risks of the study, as well as relevant information about alternative treatment. Written informed consent will be obtained. Participants shall be given sufficient time to decide whether to participate, and they have the right to withdraw at any time. In addition, informed consent is not obtained by doctors or experts in charge, and informed consent is kept in the locked filing cabinet for future reference. Personal information will be de-identified during the study.

1. **Data Confidentiality**

In accordance with the GCP guideline, the audit team should check the CRF against the original data. The informed consent should include a statement that authorize sponsor or authority agency can directly access the original data on the case report form (such as the patient's medical document, appointment records, original lab records, etc.). Investigators are expected to follow professional confidentiality rules and must keep all personally identifiable or medical information confidential.

The ownership and management of intellectual property rights are in accordance with the relevant laws and regulations of Beijing Hospital Administration Center. The research paper should be published labeling Beijing Municipal Administration of Hospitals Incubating Program (PX2022018).

**Investigators**

| **Name** | **Professional title/profession** | **assignment** | **Year of GCP training** |
| --- | --- | --- | --- |
| Jia Dong | Associate consultant /  Anesthesia | Intervention | 2017 |
| Juan Wang | Attending /  Anesthesia | Intervention | 2020 |
| Jie Wang | Resident /Anesthesia | Intervention | 2017 |
| Min Zeng | Associate consultant /Anesthesia | Intervention/Quality control | 2020 |
| Muhan Li | Resident /Anesthesia | Intervention | 2017 |
| Yuming Peng | Consultant/Anesthesia | Research design /Quality control | 2020 |

1. **Reference**

1. Baldi, I., et al., *Epidemiology of meningiomas.* Neurochirurgie, 2018. **64**(1): p. 5-14.

2. Rajagopalan, V., et al., *Effect of Intraoperative Blood Loss on Perioperative Complications and Neurological Outcome in Adult Patients Undergoing Elective Brain Tumor Surgery.* J Neurosci Rural Pract, 2019. **10**(4): p. 631-640.

3. Goh, K.Y., et al., *Tissue plasminogen activator expression in meningiomas and glioblastomas.* Clin Neurol Neurosurg, 2005. **107**(4): p. 296-300.

4. Brandel, M.G., et al., *Impact of preoperative endovascular embolization on immediate meningioma resection outcomes.* Neurosurg Focus, 2018. **44**(4): p. E6.

5. Hooda, B., et al., *Effect of tranexamic acid on intraoperative blood loss and transfusion requirements in patients undergoing excision of intracranial meningioma.* J Clin Neurosci, 2017. **41**: p. 132-138.

6. Kisilevsky, A., et al., *Anaemia and red blood cell transfusion in intracranial neurosurgery: a comprehensive review.* Br J Anaesth, 2018. **120**(5): p. 988-998.

7. Glance, L.G., et al., *Association between Intraoperative Blood Transfusion and Mortality and Morbidity in Patients Undergoing Noncardiac Surgery.* Anesthesiology, 2011. **114**(2): p. 283-292.

8. Hill, G.E., et al., *Allogeneic Blood Transfusion Increases the Risk of Postoperative Bacterial Infection: A Meta-analysis.* Journal of Trauma and Acute Care Surgery, 2003. **54**(5).

9. Ortmann, E., M.W. Besser, and A.A. Klein, *Antifibrinolytic agents in current anaesthetic practice.* Br J Anaesth, 2013. **111**(4): p. 549-63.

10. Behmanesh, B., et al., *Efficacy of Intraoperative Blood Salvage in Cerebral Aneurysm Surgery.* J Clin Med, 2021. **10**(24).

11. Henry, D.A., et al., *Anti-fibrinolytic use for minimising perioperative allogeneic blood transfusion.* Cochrane Database Syst Rev, 2007(4): p. Cd001886.

12. Henry, D.A., et al., *Anti-fibrinolytic use for minimising perioperative allogeneic blood transfusion.* Cochrane Database Syst Rev, 2011(1): p. Cd001886.

13. Adler Ma, S.C., et al., *Tranexamic acid is associated with less blood transfusion in off-pump coronary artery bypass graft surgery: a systematic review and meta-analysis.* J Cardiothorac Vasc Anesth, 2011. **25**(1): p. 26-35.

14. Molenaar, I.Q., et al., *Efficacy and safety of antifibrinolytic drugs in liver transplantation: a systematic review and meta-analysis.* Am J Transplant, 2007. **7**(1): p. 185-94.

15. Huang, F., et al., *The use of tranexamic acid to reduce blood loss and transfusion in major orthopedic surgery: a meta-analysis.* J Surg Res, 2014. **186**(1): p. 318-27.

16. Murkin, J.M., et al., *High-dose tranexamic Acid is associated with nonischemic clinical seizures in cardiac surgical patients.* Anesth Analg, 2010. **110**(2): p. 350-3.

17. Lecker, I., et al., *Tranexamic acid-associated seizures: Causes and treatment.* Ann Neurol, 2016. **79**(1): p. 18-26.

18. Hemapriya, L., G. More, and A. Kumar, *Efficacy of Tranexamic Acid in Reducing Blood Loss in Lower Segment Cesearean Section: A Randomised Controlled Study.* J Obstet Gynaecol India, 2020. **70**(6): p. 479-484.

19. Ker, K., et al., *Avoidable mortality from giving tranexamic acid to bleeding trauma patients: an estimation based on WHO mortality data, a systematic literature review and data from the CRASH-2 trial.* BMC Emerg Med, 2012. **12**: p. 3.

20. Shakur, H., et al., *Effects of tranexamic acid on death, vascular occlusive events, and blood transfusion in trauma patients with significant haemorrhage (CRASH-2): a randomised, placebo-controlled trial.* Lancet, 2010. **376**(9734): p. 23-32.

**Study protocol Modify Description**

| Modify item/page/line number | Original contents- the protocol for ethics review (V1.1.1) | Modified contents-the protocol manuscript |
| --- | --- | --- |
| Study method-standard anesthesia management/Page10/line10 | Intraoperative anesthesia can be maintained by intravenous anesthesia or combined with intravenous anesthesia, | Anesthesia will be then maintained with combined intravenous anesthesia and inhalational anesthesia. |
| Study method-standard anesthesia management/Page10/line11 | and the intraoperative BIS will be maintained between 40 and 60. | to maintain the BIS value between 40 and 50. |
| Study method-standard anesthesia management/Page10/line26 | H3 receptor blocker (Tropisetron 5mg) will be administered before end of surgery to prevent nausea and vomiting. | Not mentioned. |
| Study method-statistics analysis for primary outcome /page15/line26 | The Student’s t test for primary outcome analysis | Student t test or the Mann-Whitney U test, the multiple linear regression model or two-way analysis of covariance will be applied to account for potential confounders. |
| Study method-statistic analysis for missing data/page15 | Not mentioned | Missing data will be imputed using the worst-case scenarios and last observation carry forward method. |
| Study method-statistic analysis/page4/line14 | Event time data will be analyzed using Kaplan-Meier survival analysis, and differences among groups were assessed using log-rank test and proportional risk regression models. | Not time-to-event analysis. |
| Secondary Outcome assessments/Page19/line20 | Calculated blood loss: Calculated blood loss = estimated blood volume×(Hcti−Hctf)+transfused RBC volume]/Hctmean. | Calculated blood loss is assessed by the formula：  Calculated blood loss (CBL) = estimated blood volume×(Hcti−Hctf)+transfused RBC volume]/Hctmean  Estimated blood volume for women=[weight (kg)^0.425 × height (cm)^0.725 ] × 0.007184 × 2217+age (years) × 106  Estimated blood volume for men =[weight(kg)^0.425 x height (cm)^0.725 ]× 0.007184 x 3,064-825 |
| Intervention/page9/line25 | TXA continuous infusion of 5mg/kg/h until the end of the operation | TXA continuous infusion of 5mg/kg/h until dural closure |
