# Supplementary material for: The effect of tranexamic acid on intraoperative blood loss in patients undergoing brain meningioma resections: Study protocol for a randomized controlled trial
Source: PLoS One. 2023 Aug 31;18(8):e0290725. doi: 10.1371/journal.pone.0290725 (PMC10470952; doi:10.1371/journal.pone.0290725)
Supplement: S3 File — (DOC) [file pone.0290725.s003.doc]

项目来源与编号： PX-2022018

**巨大脑膜瘤切除术患者应用氨甲环酸的有效性研究**

所属项目名称：巨大脑膜瘤切除术患者应用氨甲环酸的有效性研究

项目负责人/承担科室：李姝/首都医科大学附属北京天坛医院麻醉科

课题委托单位：北京市医院管理中心

课题承担单位：首都医科大学附属北京天坛医院

课题主持单位：首都医科大学附属北京天坛医院

研究年限：2022年01月－ 2024年12月

版本号：V1.1.1

版本日期：2022年05月09日

**方 案 摘 要**

| **项目名称** | 巨大脑膜瘤切除术患者应用氨甲环酸的有效性研究 |
| --- | --- |
| **研究目的** | 1、探讨氨甲环酸对巨大脑膜瘤切除术中出血量的影响  2、探讨氨甲环酸对巨大脑膜瘤切除术中患者凝血功能、术野血液回收、异体血液制品使用的影响；探讨氨甲环酸对巨大脑膜瘤切除程度及切除后术野渗血的影响；  3、探讨氨甲环酸对巨大脑膜瘤切除术患者术后癫痫及其他不良事件的影响；  4、探讨氨甲环酸对巨大脑膜瘤切除术后患者住院时长、ICU住院及住院费用的影响。 |
| **研究设计** | 前瞻性、单中心、随机、对照、盲法试验 |
| **病例总数** | 228例 |
| **病例选择** | 入选标准 ***择期行巨大脑膜瘤切除术患者，术前影像学（CT或MR）提示肿瘤直径大于5cm；*** 2）18-65岁患者；  3）ASA I~III级患者；  4）获得书面的知情同意书。 |
|  | 排除标准   1. 既往氨甲环酸过敏史； 2. 既往血栓栓塞疾病史； 3. 正在接受抗凝治疗。 |
| **治疗方案** | 确认患者符合入选标准后，将患者随机分配至3个试验组之一:高剂量组、低剂量组以及对照组；其中高剂量组患者在麻醉诱导后10分钟内给予20mg/kg负荷剂量氨甲环酸，然后以5 mg/kg/h的速率持续输注；低剂量组患者在10分钟内接受负荷剂量氨甲环酸20mg/kg，后持续输注等容量生理盐水；对照组患者给予等容量生理盐水。在术中、术后1-5天以及6个月进行随访，收集数据。 |
| **疗效评定** | 主要疗效指标：估算术中失血量  次要疗效指标：  1）术中凝血功能监测；  2）计算术中失血量；  3）术中血液回吸收、异体血液制品的使用率及用量；  4）肿瘤切除后术野渗血评分。 |
|  | 安全性指标：   1. 术后癫痫发作频率和次数； 2. 其他术中并发症：过敏、低氧血症、难治性低血压、肺栓塞等 3. 术后其他并发症：静脉血栓症、新发脑缺血、颅内血肿、脑积水、感染、肾功能异常、心肌梗死。 |
|  | 经济学指标：   1. ICU入住率，ICU监护时长； 2. 住院时长及住院费用。 |
| **统计方法** | 主要结局指标：独立样本t检验。 次要结局指标：正态分布计量资料数据以均数±标准（mean±SD）表示，非正态分布数据以中位数及四分位数（median，IQR）表示。使用独立样本t检验比较正态分布的连续数据，使用独立样本Mann–Whitney U检验比较非正态分布连续数据。分类数据采用X检验或连续性校正X检验进行比较。用Kaplan-Meier生存分析分析事件发生时间数据，用对数秩检验以及比例风险回归模型评估各组间的差异。 |
| **研究期限** | 2022年1月-2024年12月 |

**一、研究背景**

脑膜瘤为最常见的颅内肿瘤，约占颅内肿瘤的30%以上[1]。虽然脑膜瘤肿瘤性质多为良性，但因其占位效应，对血管的侵蚀，脑膜瘤患者常伴有严重的颅内压增高，脑水肿，颅神经损伤，从而继发严重的神经功能障碍。巨大脑膜瘤，即直径超过5cm的脑膜瘤，占颅内肿瘤的11%[2]，手术切除肿瘤占位及相关组织是其最佳治疗手段之一。但大多数巨大脑膜瘤，血供丰富，生长过程中压迫和/或扭曲血管，部分脑膜瘤还包绕周围血管累及硬脑膜静脉窦，侵犯头皮和颅骨，切除过程中出血量可高达4000mL[2]。同时，由于软脑膜富含组织纤溶酶原激活剂，诱导纤维蛋白溶解加重术中失血[3]。巨大脑膜瘤切除术中大出血及严重的血流动力学波动，可能导致终末器官损伤、凝血功能障碍甚至危及生命[4, 5]。因此巨大脑膜瘤切除术的血液管理是神经外科围术期管理的常见挑战。

神经外科手术围术期血液管理手段很多，但每一种血液管理技术都有一定的不足且经济负担较重。即使采用了以上全部的血液管理方式，仍有部分病例因失血较多而不能避免异体输血，而异体输血有引发输血免疫抑制反应、溶血、术后感染、输血相关肺损伤等风险，从而住院时间延长，甚至死亡[6-10]。

氨甲环酸（Tranexamic Acid，TXA）是一种人工合成的抗纤溶药物，结构与赖氨酸类似，TXA可通过与纤溶酶赖氨酸位点特异性结合，抑制纤溶酶与纤溶酶原结合，阻断纤溶过程，从而阻止血栓溶解，减少出血，降低输血率[11, 12]。

目前TXA已广泛用于创伤、耳鼻喉、产科、心脏外科及骨科[13-20]，TXA能否有效的减少神经外科脑膜瘤手术患者术中出血，降低血制品输注，同时不增加围术期不良事件，仍需进一步研究。既往的研究结果表明，TXA可显著降神经外科手术低出血量(830ml vs 1124ml，p=0.03)，但该研究纳入患者肿瘤位置多样，包括颅底，鞍旁，幕下，矢状窦旁等，且实验肿瘤大小不一，对于TXA减少巨大脑膜瘤切除术患者出血量尚未可知。

综合上述研究背景，提出本研究假说：巨大脑膜瘤切除术患者术中不同剂量TXA治疗可有效减少术中出血。

**二、研究目的**

**1. 主要目的：**探讨TXA对巨大脑膜瘤术中出血量的影响。

**2. 次要目的：**

1）探讨TXA对巨大脑膜瘤切除术中患者凝血功能、术野血液回收，异体血液制品使用的影响；

2）探究TXA对巨大脑膜瘤切除程度及切除后术野渗血的影响；

3）探讨TXA对巨大脑膜瘤切除术患者术后癫痫及其他不良事件的影响；

4）探讨TXA对巨大脑膜瘤切除术后患者住院时长、ICU住院及住院费用的影响。

**三、研究设计类型、原则与试验步骤**

**1. 研究设计**

1）研究设计类型：本研究是一项前瞻、随机、对照、盲法研究。

2）随机化分组方法：研究人员通过计算机软件生成随机序列，根据随机数字的分组，患者按照1:1:1随机分至3个试验组之一:高剂量组、低剂量组以及对照组。

3）设盲：本研究对参与研究的受试者、术中手术及麻醉医生、术后随访评估人员设盲。最后一名受试者所有终点事件评估结束后揭晓分组情况。

4）研究中心：首都医科大学附属北京天坛医院麻醉科。

5）样本量：228例。

6）适应症的合理性及确定依据：目前对于氨甲环酸在临床中的研究主要局限于骨科、产科、心外科、创伤科等，在神经外科领域研究较少，而巨大脑膜瘤由于肿瘤巨大，血供丰富，对血管侵蚀较严重，神经损伤较多，因此术中容易大出血。既往研究针对氨甲环酸对脑膜瘤术中失血量的影响展开随机对照研究，发现氨甲环酸显著减少了术中出血量。但研究样本量较小，肿瘤位置及类型较多，因此该研究结果无法应用于巨大脑膜瘤人群。本研究旨在进一步探讨氨甲环酸对直径超过5cm的脑膜瘤术中出血量的影响。本中心目前已经确定了氨甲环酸在脑膜瘤切除术患者中的安全性应用，因此需更深地探讨氨甲环酸对巨大脑膜瘤切除术患者的有效性。

**2. 样本量及研究计划**

根据既往研究研究报道，巨大脑膜瘤术中出血量约1000ml，人群整体标准差约为400ml。假设单次TXA输注可有效降低出血量约25%，持续TXA输注可进一步降低出血量25%，因此最小效应量为250ml，总体P=0.05，1-β=0.9，加上2.5%脱落率，得出每组76例，最终样本量总量约为228例。

**3. 研究期限**

2022年1月-2024年12月。

**4. 受试者选择**

计划于2022年1月至2024年12月在首都医科大学附属北京天坛医院连续招募择期巨大脑膜瘤切除术患者。

纳入标准：择期行巨大脑膜瘤切除术患者，术前影像学（CT或MR）提示肿瘤直径大于5cm；18-80岁患者；ASA I~III级患者；获得书面的知情同意书。

排除标准：既往TXA过敏史；既往癫痫发作病史；既往血栓栓塞疾病史；正在接受抗凝治疗；无法获得知情同意书。

当试验过程中出现了严重的不良反应，不良事件将被密切监测，直至其得到解决和稳定。一旦发生不良事件，将立即向相关部门报告，并通知主要研究者确定不良事件的严重程度和后果。如患者出现严重试验药物过敏等术中不良事件，术中麻醉医师课停止试验药物的输注。

**四、研究方法**

1. 盲法：参与研究的受试者、麻醉医生、术后随访评估者对随机分组结果盲法。随机化结果封存于不透明信封中，只有最后一名受试者所有终点事件评估结束后，才揭晓分组情况。如果受试者干预治疗过程中出现严重威胁生命的不良事件时，由主要研究者决定是否揭盲，并该病例是为脱落病例。试验用药物与安慰剂，由不知患者分组结果的单独研究人员依照随机号对应结果完成配制，并标示“试验用药”，药物配制人员不参与患者干预过程，不参与受试者干预后任何评估及随访环节。
2. 分组及干预方法：受试者随机分为三组，分别为低剂量组，高剂量组及安慰剂组（对照组）。依照随机化结果，在麻醉诱导结束后开始静脉注射不同剂量TXA或安慰剂（生理盐水）。试验药物为氨甲环酸（生产厂家： 剂量：200mg/2mL）。低剂量组单次静脉输注TXA剂量为20mg/kg；高剂量组TXA组先单次静脉输注20mg/kg TXA后持续泵注5mg/kg/h至手术结束；对照组为等容量0.9% 生理盐水。单次输注的TXA与生理盐水均保存于避光100ml容器，输注速度为250ml/h。
3. 标准化术中麻醉管理：患者入室建立可靠静脉通路，常规监测心电图、无创血压、脉氧饱和度监测、呼末二氧化碳监测及脑电双频指数监测。充分预充氧6min后，开始标准化麻醉诱导，诱导药物使用丙泊酚1-2mg/kg或依托咪酯0.2mg/kg，舒芬太尼0.2-0.4ug/kg，罗库溴铵0.9mg/kg或顺式阿曲库铵0.2mg/kg。药物实际剂量可依照患者身体情况，患者循环变化及手术耐受程度酌情调整。术中麻醉维持可选择全凭静脉麻醉或静吸复合麻醉两种麻醉方法，维持术中脑电双频指数为40-60之间。术毕前给予H3受体阻断剂（托烷司琼5mg），预防恶心呕吐。所有用药均需详细记录，手术结束后患者转运至病房或麻醉恢复室或ICU观察。术后常规使用患者自控阵痛装置，镇痛配方药物选择舒芬太尼，剂量由主管麻醉医师决定。常规术后镇痛剂量下，受试者仍存在严重术后疼痛情况（定义为：数字模拟疼痛评分≥5/10，且出现疼痛难忍躁动），可经主管医师允许下追加其他镇痛药物，但需详细记录，给药原因及给药种类。

**五、伴随用药**

本研究为临床麻醉常规，临床麻醉常规用药都可以为伴随用药，而临床麻醉禁忌或者停止用药则为不可以伴随用药。

**六、观察指标与检查时间**

筛选：1）是否符合入选标准；2）是否属于排除标准；3）知情同意；4）随机化分组。

术前：基本信息、个人及家族史、既往史、既往用药、入院查体、术前辅助检查（血常规+凝血功能+血生化）、影像学初步诊断、治疗前评估（GCS评分、Charlson合并症评分、ASA分级、Karnofsky评分、Caprini血栓风险评分）。

术中：1）平均动脉压目标区间；2）是否复合神经阻滞；3）是否应用正性肌力药或血管活性药；4）平均吸入氧浓度；5）术中凝血检查；6）术中血气检查； 7）估计出血量；8）输血种类及剂量；9）术中不良事件；10）肿瘤切除程度；11）术中渗血评估及止血难易程度；12）术中止血材料使用情况；13）麻醉药物；14）麻醉过程中药物参数；15）麻醉过程中生理参数。

PACU：1）进入PACU时间；2）PACU事件；3）PACU药物使用；4）离开PACU时间。

术后1天：1）临床结局评估（评估时间、死亡、KPS评分，GCS昏迷评分、术后癫痫、血管栓塞、感染、术后血肿、二次手术、入住ICU、死亡、其他不良事件以及患者神经功能障碍评估）；2）术后第1天实验室检查结果；3）术后第1天Caprini血栓风险评。

术后3天：临床结局评估：评估时间、死亡、术后癫痫、血管栓塞、感染、术后血肿、二次手术、入住ICU、死亡、其他不良事件以及患者神经功能障碍评估。

术后5±2天：1）临床结局评估：评估时间、死亡、术后癫痫、血管栓塞、感染、术后血肿、二次手术、入住ICU、死亡、其他不良事件以及患者神经功能障碍评估；2）术后第5±2天Brice术中知晓评估；3）术后影像学评估及脑电图监测。

出院访视：1）出院病理诊断；2）出院总结（出院时间，结局，住院天数、出院去向、ICU停留总时间、住院总花费、出院后癫痫药物）；3）术后治疗方案；4）出院临床评估（贫血、癫痫、栓塞、感染、血肿、二次手术、输血等）。

术后180访视：1）临床结局评估（评估时间、死亡、KPS评分，GCS昏迷评分、术后癫痫、血管栓塞、感染、术后血肿、二次手术、入住ICU、死亡、其他不良事件以及患者神经功能障碍评估）。

**七、随访时间**

随访时间：术前第一天、术后第1、3、5±2天、180天。

随访内容：术后第1天辅助检查（血常规+凝血功能+血生化）、术后第1天Caprini风险评分、术后第1，3，5±2，出院、180天KPS评分，GCS昏迷评分，并发症等评估。

**八、临床评价**

本研究的主要结局指标是术中出血量，计算公式为：估算手术出血量（ml）=负压吸引装置内容量+手术前后纱布棉条增加的重量-术中冲洗盐水容量。

次要结局指标包括：

1. 术中凝血功能监测：手术开始及结束时分别监测血栓弹力图监测凝血功能；
2. 计算术中失血量：计算手术出血量（ml）=体重*70*[（术前Hb+输入Hb-血中现存Hb/术前Hb）；
3. 术中血液回吸收、异体血液制品的使用率及用量；本研究中心的常规输血的指征：a. 异体红细胞输注为Hb＜9g/dl或Hct＜30%。如术中病理WHO≤II，则优先自体血液回输；缺乏术中病理或考虑WHO＞II则，优先使用异体血。b. 冰冻血浆、冷沉淀和血小板制品的使用，按照美国麻醉学家协会血液成分治疗特别工作组的建议进行治疗：术中PT/PTT延长1.5倍，纤维蛋白原<1g/L，或创缘有弥漫性微血管出血(尽管手术止血和/或手术野内无可见凝块)。
4. 肿瘤切除程度：采用Simpson脑膜瘤切除程度评分；
5. 肿瘤切除后术野渗血评分：采用Hooda评分法进行评估。
6. 止血难易程度评分：采用数字模拟量表进行评分。

安全性指标包括：1）术后癫痫发作频率和次数；2）其他术中并发症（过敏、低氧血症、难治性低血压、肺栓塞等）及术后其他并发症（静脉血栓症、新发脑缺血、颅内血肿、脑积水、感染、肾功能异常、心肌梗死）。

经济学指标：1）ICU入住率，监护时长；2）住院时长及住院费用。

**九、项目已知和潜在的风险和获益，以及对风险的处置预案**

从输注研究药物开始到术后第五天，研究者将密切监测氨甲环酸的不良反应。研究人员将记录所有不良反应，包括类型、发生时间、持续时间以及预后。术中责任麻醉师有权力停止输注研究药物并记录原因。研究人员将密切监测所有不良事件，直到达到稳定状态。主要研究者将被告知任何严重不良事件，并确定这些事件的严重性和因果关系。所有与研究相关的不良事件都将被记录并作为年度报告的一部分上交给伦理委员会。主要研究者将对不良事件负责。

根据文献报道，氨甲环酸主要的不良反应为癫痫，其次为栓塞性事件，当出现相应的不良反应后，需及时采取应急措施，如镇静药控制癫痫，扩血管药物与补液治疗减少缺血性事件发生等。

**十、研究的质量控制与质量保证**

1. 研究者职责

研究者保证在进行临床试验时遵循临床试验方案，遵循药品临床试验管理规范以及相应法律法规。

研究者应保证遵循临床试验方案中的所有研究操作（包括安全性原则）。研究者应根据相关要求，以一种准确、清晰的方式正确提供可靠数据和临床试验方案要求的所有信息，并确保检查团队可直接查看原始资料。

研究者可能会任命他/她认为合适的人作为协助研究者。协助研究者将根据临床试验方案协助进行临床试验的管理。所有的协助研究者将被及时任命并记录。协助研究者接受研究者的监督和管理。研究者将提供给他们一份临床试验方案和所有必需的信息。临床试验的申办方对卫生当局负责，通过采取所有适当的手段来确保临床试验的合理管理，这些手段如伦理、临床试验方案依从性、记录在病例报告表中数据额完整性和有效性等。

1. 研究监查

监查团队的主要职责是帮助研究者和申办方保证临床试验各个方面的高度的伦理性、科学性、专业性和规范性。根据GCP原则，监督团队应对照原始资料对CRF进行核查。

监查团队将定期通过现场访视、信件或电话形式，评估研究进展、研究者和患者对临床方案的依从性以及解决紧急的问题。在这些监查访问中，监查员将和研究者共同监查，要点如下（并非无遗漏）：患者的知情同意、患者的招募和随访、严重不良事件文件的记录和报告。

**十一、资料保存**

所有数据将记录在病例报告表中，病例报告表将保存于科内专属科研资料储存柜中至少5年。原始数据和非数字数据均被编码用于数据存储、审查、制表和分析。数据将安全地输入、存储在电子数据库中。将采用双重数据输入。

**十二、数据安全监查**

该项目将由麻醉学、伦理学、统计学和方法学专家组成的数据监测委员会（Data Monitoring Committee, DMC），作为一个独立的机构来监测试验的安全性、有效性、伦理问题和进展。DMC将通过定期访谈或电话进行审计。DMC保留随时审核患者招募的权利。审计过程将独立于调查人员。

**十三、统计学处理**

1. 样本量及统计效率：根据既往研究研究报道，巨大脑膜瘤术中出血量约1000ml，人群整体标准差约为400ml。单次TXA输注可有效降低出血量约25%，持续TXA输注可进一步降低出血量25%，因此最小效应量为250ml，总体P=0.05，1-β=0.9，加上2.5%脱落率，得出每组76例，最终样本量总量约为228例。
2. 统计分析策略：基于意向性分析分析（Intention-to-treat）进行报道。
3. 主要结局指标 采用Student t检验进行组间比较，p<0.017认为两组间差异有显著性。总体p<0.05认为三组出血量有显著差异。
4. 次要结局指标 正态分布资料组间比较使用Student t检验，非正态分布资料使用Mann–Whitney U检验。计数资料组间比较使用卡方检验。研究主要结局指标术中失血量为连续变量，采用Student t检验或Mann-Whitney U检验进行差异性检验。组间比较采用Bonferroni法校正P<0.017为差异有统计学意义。

**十四、伦理考虑**

临床研究将遵循世界医学大会《赫尔辛基宣言》等相关规定。在研究开始之前，由伦理委员会批准该试验方案后才实施临床研究。每一位受试者入选本研究前，研究者有责任向受试者或其合法代理人全面地介绍本研究的目的、程序和可能的风险，以及替代治疗的相应信息，并签署书面知情同意书，应给予受试者充足的时间考虑，让受试者知道他们有权随时退出本研究，并且不由主管医生或者专家进行知情告知，知情同意书作为临床研究文件保留备查。研究过程中将保护受试者的个人信息不被泄露。

**十五、资料保密**

根据GCP 原则，监督团队应对照原始资料来核查 CRF。知情同意书将包括一个声明，即患者允许已授权的申办方、权威机构直接查阅病例报告表上相关的原始资料(如患者的医疗档案、预约记录、原始实验室记录等)。研究人员应遵循职业保密规定，必须对患者的所有个人身份信息或医疗信息保密。

知识产权的归属和管理按照课题国家有关法律法规及课题主管部门-北京市医院管理中心规定执行。研究论文发表标注北京市医院管理中心培育项目及项目号PX2022018。

**十六、参加人员**

| **姓名** | **职称/专业** | **任务** | **GCP培训（时间）** |
| --- | --- | --- | --- |
| 董佳 | 副主任医师/麻醉 | 研究实施 | 2017 |
| 王娟 | 主治医师/麻醉 | 研究实施 | 2020 |
| 王洁 | 住院医师/麻醉 | 研究实施 | 2017 |
| 曾敏 | 副主任医师/麻醉 | 研究实施，质量控制 | 2020 |
| 李沐寒 | 住院医师/麻醉 | 研究实施 | 2017 |
| 彭宇明 | 主任医师/麻醉 | 研究设计，质量控制 | 2020 |

**十七、参考文献**

1. Baldi, I., et al., *Epidemiology of meningiomas.* Neurochirurgie, 2018. **64**(1): p. 5-14.

2. Rajagopalan, V., et al., *Effect of Intraoperative Blood Loss on Perioperative Complications and Neurological Outcome in Adult Patients Undergoing Elective Brain Tumor Surgery.* J Neurosci Rural Pract, 2019. **10**(4): p. 631-640.

3. Goh, K.Y., et al., *Tissue plasminogen activator expression in meningiomas and glioblastomas.* Clin Neurol Neurosurg, 2005. **107**(4): p. 296-300.

4. Brandel, M.G., et al., *Impact of preoperative endovascular embolization on immediate meningioma resection outcomes.* Neurosurg Focus, 2018. **44**(4): p. E6.

5. Hooda, B., et al., *Effect of tranexamic acid on intraoperative blood loss and transfusion requirements in patients undergoing excision of intracranial meningioma.* J Clin Neurosci, 2017. **41**: p. 132-138.

6. Kisilevsky, A., et al., *Anaemia and red blood cell transfusion in intracranial neurosurgery: a comprehensive review.* Br J Anaesth, 2018. **120**(5): p. 988-998.

7. Glance, L.G., et al., *Association between Intraoperative Blood Transfusion and Mortality and Morbidity in Patients Undergoing Noncardiac Surgery.* Anesthesiology, 2011. **114**(2): p. 283-292.

8. Hill, G.E., et al., *Allogeneic Blood Transfusion Increases the Risk of Postoperative Bacterial Infection: A Meta-analysis.* Journal of Trauma and Acute Care Surgery, 2003. **54**(5).

9. Ortmann, E., M.W. Besser, and A.A. Klein, *Antifibrinolytic agents in current anaesthetic practice.* Br J Anaesth, 2013. **111**(4): p. 549-63.

10. Behmanesh, B., et al., *Efficacy of Intraoperative Blood Salvage in Cerebral Aneurysm Surgery.* J Clin Med, 2021. **10**(24).

11. Henry, D.A., et al., *Anti-fibrinolytic use for minimising perioperative allogeneic blood transfusion.* Cochrane Database Syst Rev, 2007(4): p. Cd001886.

12. Henry, D.A., et al., *Anti-fibrinolytic use for minimising perioperative allogeneic blood transfusion.* Cochrane Database Syst Rev, 2011(1): p. Cd001886.

13. Adler Ma, S.C., et al., *Tranexamic acid is associated with less blood transfusion in off-pump coronary artery bypass graft surgery: a systematic review and meta-analysis.* J Cardiothorac Vasc Anesth, 2011. **25**(1): p. 26-35.

14. Molenaar, I.Q., et al., *Efficacy and safety of antifibrinolytic drugs in liver transplantation: a systematic review and meta-analysis.* Am J Transplant, 2007. **7**(1): p. 185-94.

15. Huang, F., et al., *The use of tranexamic acid to reduce blood loss and transfusion in major orthopedic surgery: a meta-analysis.* J Surg Res, 2014. **186**(1): p. 318-27.

16. Murkin, J.M., et al., *High-dose tranexamic Acid is associated with nonischemic clinical seizures in cardiac surgical patients.* Anesth Analg, 2010. **110**(2): p. 350-3.

17. Lecker, I., et al., *Tranexamic acid-associated seizures: Causes and treatment.* Ann Neurol, 2016. **79**(1): p. 18-26.

18. Hemapriya, L., G. More, and A. Kumar, *Efficacy of Tranexamic Acid in Reducing Blood Loss in Lower Segment Cesearean Section: A Randomised Controlled Study.* J Obstet Gynaecol India, 2020. **70**(6): p. 479-484.

19. Ker, K., et al., *Avoidable mortality from giving tranexamic acid to bleeding trauma patients: an estimation based on WHO mortality data, a systematic literature review and data from the CRASH-2 trial.* BMC Emerg Med, 2012. **12**: p. 3.

20. Shakur, H., et al., *Effects of tranexamic acid on death, vascular occlusive events, and blood transfusion in trauma patients with significant haemorrhage (CRASH-2): a randomised, placebo-controlled trial.* Lancet, 2010. **376**(9734): p. 23-32.
