# Supplementary material for: The effect of tranexamic acid on intraoperative blood loss in patients undergoing brain meningioma resections: Study protocol for a randomized controlled trial
Source: PLoS One. 2023 Aug 31;18(8):e0290725. doi: 10.1371/journal.pone.0290725 (PMC10470952; doi:10.1371/journal.pone.0290725)
Supplement: S1 Table — (DOCX) [file pone.0290725.s004.docx]

**S1 Table. Definition of complications**

| **Complications** | **Definition** |
| --- | --- |
| Drug allergy | According to the international consensus on drug allergy[1]: Ⅰ:only presenting with skin flushing, maculopapules and hives; Ⅱ:in addition to skin symptoms, hypotension, tachycardia, dyspnea and gastrointestinal symptoms; Ⅲ:skin symptoms; tachycardia or bradycardia arrhythmia; bronchospasm and gastrointestinal symptoms; Ⅳ:cardia arrest |
| Intraoperative hypoxemia | Defined as intraoperative oxygen saturation is below 90% with pure oxygen inhalation |
| Refractory hypotension | Defined as MAP<65mmHg with accumulative duration >15min |
| Massive hemorrhage | Defined as loss of 100% circulating blood volume within 1 hour or loss of at least 50% circulating blood volume within 3 hours with bleeding rate of 150mL/min or 1.5mL/kg/min over 20min |
| Seizure | According to the international league against epilepsy: 1.focal seizure: limited to the unilateral cerebral hemisphere, manifested as unilateral seizure symptoms; 2.generalized seizure: bilateral cerebral hemisphere involvement, manifested as bilateral seizure symptoms; ①absence seizure (also known as small attacks): a sudden gaze with a disturbance of consciousness; ②clonic seizure: rhythmic twitch muscle contraction, usually involving the arm, neck, and face; ③myoclonic seizure: a sudden and transient muscle contraction, usually no disturbance of consciousness; ④tonic seizure: a sudden muscle stiffness, often accompanied by disturbance of consciousness; ⑤atonic seizure: a sudden loss of control of the muscles, especially the legs, leading to collapse on the ground |
| Anemia | According to the WHO[2], postoperative anemia will be classified as:  mild anemia (Hb 110-129 g/L for men; 110-119 g/L for women); moderate (Hb 80-109 g/L); severe (Hb <80 g/L) |
| Deep vein thrombosis | Patients were not routinely screened for VTE, imaging was only obtained based on clinical suspicion. Defined as clinical symptoms and the thrombosis indicated by vascular ultrasound, accompanied by signs of limb swelling or not |
| Pulmonary embolism | Indicated by pulmonary computed tomography (CTA)/MRI angiography, accompanied by clinical symptoms and signs (chest pain, shortness of breath, and hemoptysis) |
| Intracranial hematoma | Defined as postoperative neurological deficit and brain parenchyma, tumor cavity, subdural blood accumulation indicated by CT/MRI imaging confirmed by an attending neurologic consultant |
| Hydrocephalus | Defined as postoperative symptoms of intracranial hypertension, associated with CT suggestive of ventricle enlargement confirmed by the neurosurgeon |
| Infection | Defined as clinical symptoms (fever and(or) neck stiffness) , purulent secretion, positive secretion culture |
| Acute myocardial injury | According to ESC/AHA Task Force 2018[3]： Defined as elevated cardiac troponin values are 99th percentile upper reference limit and a rise and/or fall of cTn values after operation, when associated with the appearance of (1) new pathological Q waves or new left bundle-branch block, (2) angiographically documented new graft or new native coronary artery occlusion, or (3) imaging evidence of new loss of viable myocardium or new regional wall motion abnormality. In the standardized follow-up questionnaire, the related clinical symptoms and signs include chest tightness or pain, dyspnea, cyanosis, hemoptysis, and hacking cough. If a patient presents with any of these symptoms or signs, we obtain troponin levels, an electrocardiogram, and an echocardiogram. Coronary computed tomography angiography (CTA) or coronary angiography may be subsequently performed. |
| Acute kidney injury | According to the KDIGO 2012 criteria[4]，those with an increase in sCr ≥0.3mg/dL within 48 hours of surgery, or a ≥1.5 times increase from baseline within 7 days after surgery |
| Ischemic stroke | Defined as a new focal neurologic deficit confirmed by cerebral computed tomography and an attending neurologic consultant. In the standardized follow-up questionnaire, the related clinical symptoms and signs include sudden unilateral hemiplegia, sudden syncope, sudden obnubilation, and sudden amaurosis. If a patient has any of these symptoms or signs, a highly specific examination (either cerebral computed tomography or magnetic resonance imaging) is performed. |
| Mortality | Defined as death due to any cause within hospital after operation |

WHO, World Health Organization; Hb, haemoglobin; VTE, venous thromboembolism; CT, computerized tomography; MRI, Magnetic resonance imaging; ESC, European Society of Cardiology; AHA, American Heart Association; KDIGO, Kidney Disease Improving Global Outcomes.

Reference

1. Wise, S.K., et al., International Consensus Statement on Allergy and Rhinology: Allergic Rhinitis. Int Forum Allergy Rhinol, 2018. **8**(2): p. 108-352.

2. Muñoz, M., et al., An international consensus statement on the management of postoperative anaemia after major surgical procedures. Anaesthesia, 2018. **73**(11): p. 1418-1431.

3. Thygesen, K., et al., Fourth Universal Definition of Myocardial Infarction (2018). J Am Coll Cardiol, 2018. **72**(18): p. 2231-2264.

4. Khwaja, A., KDIGO clinical practice guidelines for acute kidney injury. Nephron Clin Pract, 2012. **120**(4): p. c179-84.
